# Anterior Half of the Peroneus Longus Tendon Combined with Semitendinosus and Gracilis Tendons for Anterior Cruciate Ligament Reconstruction: An Athlete Case Report

**DOI:** 10.1155/2021/9978383

**Published:** 2021-06-24

**Authors:** Diego Escudeiro de Oliveira, Melanie Mayumi Horita, Marconde de Oliveira e Silva, Victor Eduardo Roman Salas, Pedro Baches Jorge

**Affiliations:** Departamento de Ortopedia e Traumatologia, Irmandade da Santa Casa de Misericórdia de São Paulo, Brazil

## Abstract

In clinical practice, it is observed that the hamstring tendon graft, despite being first choice in knee ligament reconstruction, may not present adequate size. Therefore, it becomes necessary to search for other graft alternatives. In this context, the peroneus longus tendon arises as an option to replace or complement other grafts. The surgeon can opt to use the tendon in its totality or only its anterior half, presenting adequate length, diameter, and biomechanics, without major repercussions for the donor site. In this study, we report a case of an athlete in which the autologous hamstring tendon graft did not present the adequate diameter for anterior cruciate ligament reconstruction. It was, then, necessary to use the anterior half of the peroneus longus tendon.

## 1. Introduction

Because of the lower frequency of anterior pain and extension restriction complaints, the majority of the Anterior Cruciate Ligament (ACL) reconstructions are performed with the use of the gracilis (G) and semitendinosus (ST) tendon grafts. On the other hand, some of these grafts do not exceed 8.0 mm of diameter, thus presenting higher risk of failure [1]. Recently, the peroneus longus tendon (PL) has become a viable option of graft for knee ligament reconstruction [2]. It can be used in its totality [3–5] or only its anterior half [6, 7].

In this case report, we used a combination of G and ST and the anterior half of PL (AHPL) for ACL reconstruction in a soccer athlete that presented a G+ST graft with 7.0 mm of diameter. We obtained patient consent to publish the case and the images. The study was approved by the ethics committee of the institution with the protocol number 34826120.5.0000.5479, and the SCARE criteria were used for its formulation.

## 2. Case Presentation

A 21-year-old male patient (57kgs, 1.65 m height) attended our sports trauma ambulatory complaining about left knee pain and instability after rotational trauma during soccer practice. Physical examination showed Lachman, Anterior Drawer, and Pivot Shift test positives for ACL lesion, which was confirmed by magnetic resonance imaging. The patient was submitted to ACL reconstruction.

## 3. Surgical Technique

The surgery was performed with the patient in the supine position, under spinal anesthesia and sedation. A pneumatic tourniquet was placed on the left thigh and inflated after limb exsanguination. The procedure was accomplished by the senior surgeon of the team. Standardized anterolateral and anteromedial arthroscopic portals were used. After joint inventory, ACL lesion was confirmed.

### 3.1. Harvest

A longitudinal incision of approximately 3 cm was made, distal to the knee joint, on the pes anserinus site. The fascia of the sartorius muscle and the ST and G tendons were identified. A transverse incision was made in the sartorius fascia. ST and G tendons are repaired with No. 1.0 Vicryl thread (Ethicon, Somerville, NJ), and its proximal insertions are removed with the aid of a tenotome.

**Figure 1:**
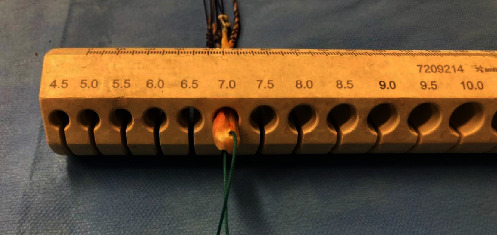
ST and G tendons folded to form a single quadruple graft, presenting 7.0 mm of diameter.

ST and G tendons were folded to form a single quadruple graft, presenting 7.0 mm of diameter and 11 cm of length (Figure 1). To augment the graft, we opted to use the AHPL.

**Figure 2:**
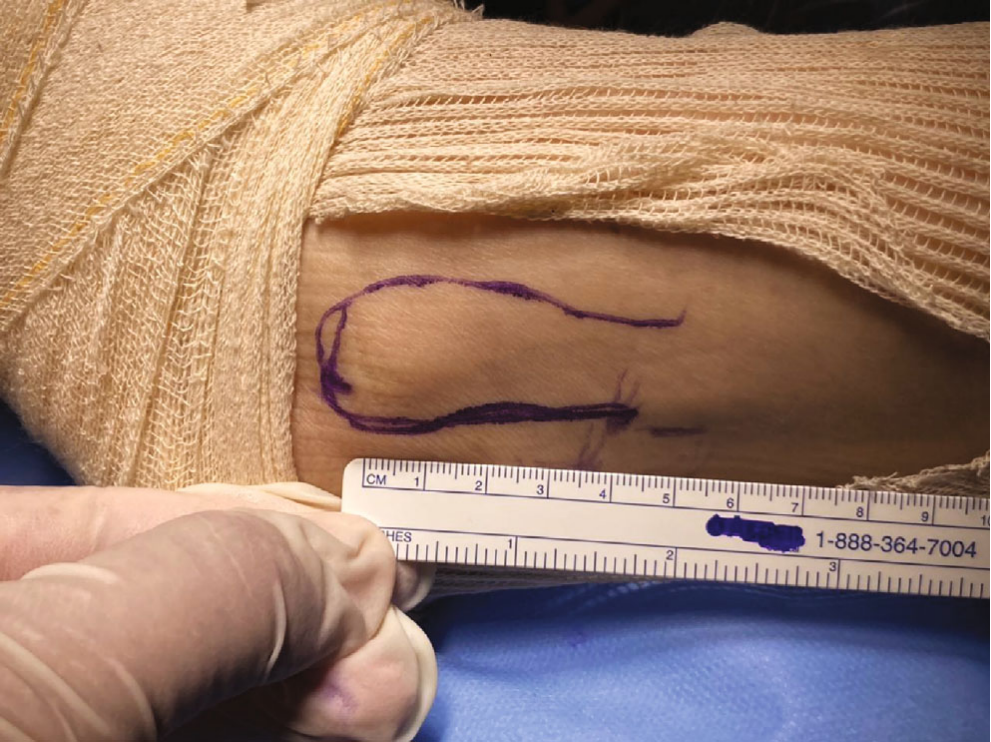
The incision started 3 cm proximal to the most distal point of the lateral malleolus and was extended 3 cm proximally.

**Figure 3:**
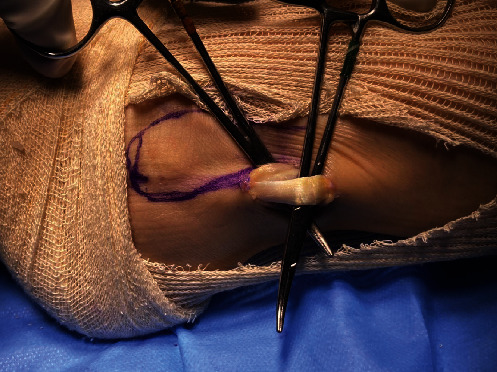
The peroneus longus was identified and distinguished from the peroneus brevis.

**Figure 4:**
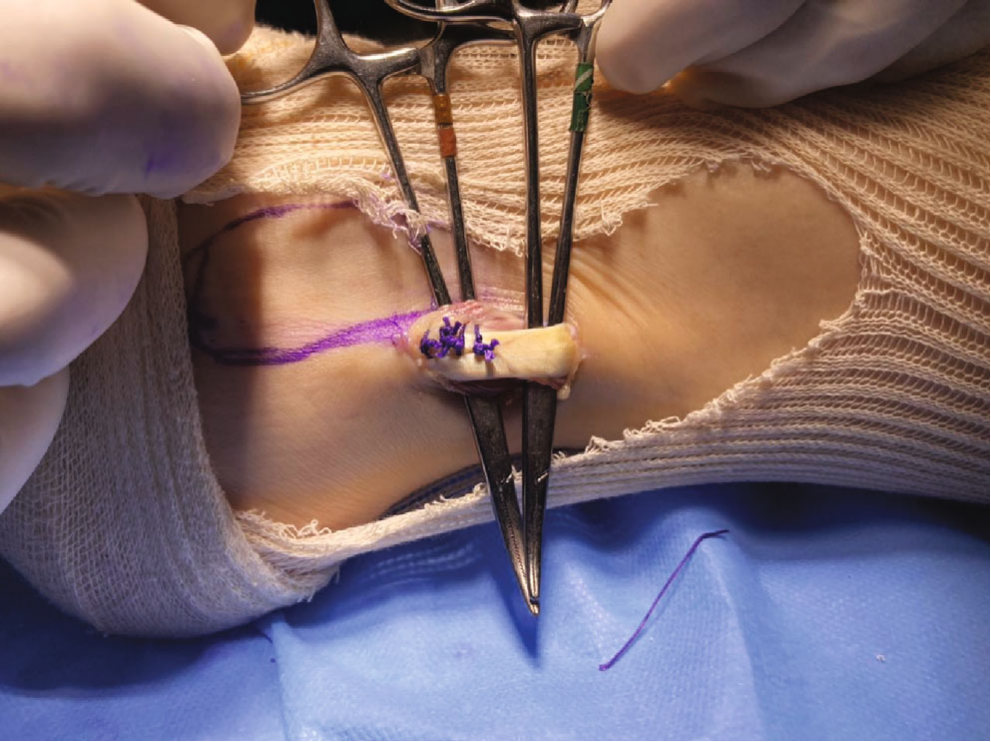
Both peroneal tendons were brought together in the most distal region of the incision.

A single longitudinal incision of approximately 3 cm was made in the posterolateral region of the fibula over the PL. The incision started 3 cm proximal to the most distal point of the lateral malleolus (Figure 2). The subcutaneous tissue was separated, and the PL was identified and isolated with the aid of a hemostatic forceps (mosquito or Kelly) after it was distinguished from the peroneus brevis (Figure 3). Both peroneal tendons were then brought together in the most distal region of the incision using single sutures with No. 1-0 Vicryl thread (Ethicon, Somerville, NJ) (Figure 4). After the tendons were unified with sutures, the PL was incised in “L” shape to withdraw its anterior half and repaired with No. 1-0 Vicryl thread (Ethicon, Somerville, NJ). It was then removed to its proximal insertion with the aid of a tenotome, up to approximately 5 cm from the fibular head, avoiding any injury to the fibular nerve.

**Figure 5:**
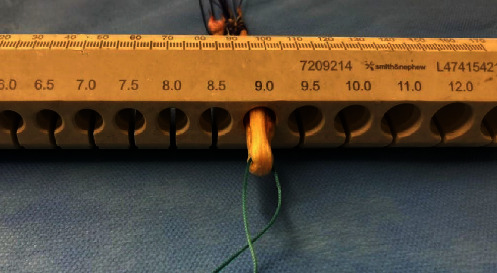
The peroneus longus graft was incorporated into the ST+G grafts forming a sixfold graft with 9.0 mm of diameter.

The PL graft was incorporated into the ST+G grafts forming a sixfold graft with 9.0 mm of diameter and 15 cm length (Figure 5).

Reconstruction was performed through anatomic positioning to create the femoral and tibial tunnels, and guides were used to make them. Initially, the femoral tunnel is performed (tunnel for button passage followed by graft tunnel) using transportal technique. After that, the tibial tunnel was made. The sixfold graft, positioned on the adjustable loop button (ULTRABUTTON Adjustable Fixation Device; Smith & Nephew), was passed through the tunnels. After loop adjustment and graft positioning check, tibial fixation was made with interference screw (Biosure; Smith & Nephew).

## 4. Evaluation

The patient was assessed before and after the procedure for knee pain with Visual Analogic Scale (VAS), and subjective evaluation with International Documentation Committee (IKDC) score and Lysholm Knee Scoring Scale was performed. The patient was also submitted to Foot and Ankle Disability Index Score (FADI) and American Orthopaedic Foot and Ankle Society Ankle-Hindfoot Scale (AOFAS) to evaluate modifications on the PL donor site.

## 5. Results

Previously to the surgery, the scores mentioned above were measured. The patient presented 47.1% on IKDC, 47% on Lysholm, 9 on VAS, and 100% on AOFAS and FADI. On physical examination, Lachman Test 3+/4+, Anterior Drawer Test 3+/4+, and Pivot Shift Test 3+/4+ were presented. The patient attended the appointments with no complaints and was submitted to physical rehabilitation. After three months of follow-up, the scores were applied again. The results were as follows: IKDC 63%, Lysholm 95%, VAS 8, and AOFAS and FADI 100% (Table 1). The patient presented progressive improvement of knee pain and function and presented no complaints about the donor ankle. On physical examination, Lachman and Anterior Drawer Tests were negative and Pivot Shift Test was 1+/4+.

## 6. Discussion

The hamstring tendons are the first choice of graft for ACL reconstruction and usually have an adequate size. However, in clinical practice, it was observed in some cases that the size of the graft might not be sufficient [7], making necessary the pursuit for new alternatives. There are evidences that weight, height, and body mass index can help predict the graft size, once individuals with lower height and weight tend to have lower hamstring diameter [6]. That enables the surgeon to use a previous surgery plan considering the use of other types of graft. Some studies indicate that the minimal diameter of the graft to avoid failure after reconstruction must be 7.0 mm [8]. Therefore, it becomes necessary to find grafts that replace or complement the grafts already established. One alternative is the use of extensor mechanism tendons that, although widely used, are not adequate for some techniques besides being able to cause anterior knee pain and thigh musculature hypotrophy [4]. Another alternative is the use of homologous grafts from tissue banks which are a good option considering the flexibility of shape and size yet, having higher cost, are not available in all institutions besides having the risk of immunologic reaction and disease transmission.

The PL tendon arises thus as a viable choice of graft for knee ligament reconstructions. Zhao and Huangfu published a cadaveric study in 2012, showing that load to rupture of PL is similar to ST and higher than G, proving that it is a good option from the biomechanical point of view, to complement or replace the hamstring graft [9]. There are many studies in the literature evidencing good results of PL use in ACL reconstruction, involving the operated knee and the donor ankle in short- and long-term evaluations [3, 4, 10–13]. Nevertheless, due to the importance of PL on foot and ankle function (plantar flexion, eversion, and transverse arch maintenance), its complete withdrawal may have a functional impact. Trying to minimize its negative impact on the donor ankle, some studies opted for its partial use. On Zhao and Huangfu's pilot study, in 2011, it was concluded that the anterior fibers of PL tendon are longer than the posterior fibers, one of the reasons why the anterior half was chosen for harvest [9]. Furthermore, the PL tendon is well vascularized by vessels that penetrate the peritenon through a posterolateral bind, making the preservation of the posterior half good for the tendon regeneration, proved by MRI and histological analysis [14]. Studies that used the AHPL did not observe meaningful changes on the assessed parameters such as AOFAS, FADI, and isokinetic evaluation, with no loss of strength or range of motion of the donor ankle [5, 7, 15].

For a graft to be considered ideal, it must have enough resistance, be easily and safely harvested, with no functional impairment to the donor site [15]. The AHPL is therefore an alternative graft with adequate tensile resistance, easy harvesting due to its superficial positioning, and absence of fibrous connections with nearby structures, besides having sufficient length and diameter. For this reason, this graft was chosen for the case described above in which the hamstring tendons were not enough for ACL reconstruction and must be considered a good option in similar cases, complementing or replacing other kinds of grafts.

## Figures and Tables

**Table 1:** Results of preoperative and postoperative evaluations.

	Preoperative	3 months after surgery
IKDC	47.1%	63%
Lysholm	47%	95%
VAS	9	8
AOFAS	100%	100%
FADI	100%	100%
Lachman Test	3+/4+	—
Anterior Drawer Test	3+/4+	—
Pivot Shift Test	3+/4+	1+/4+
